# From Effusion to Diagnosis: Integrating the FIP Effusion Index and Cell Tube Block Immunohistochemistry in a Geriatric Cat

**DOI:** 10.3390/ani16213360

**Published:** 2026-10-24

**Authors:** Ricardo Lopes, Cátia Fernandes, Hugo Lima de Carvalho, Gonçalo Barros, Filipe Sampaio

**Affiliations:** 1 CEDIVET Veterinary Laboratories, Lionesa Business Hub, R. Lionesa 446 C24, 4465-671 Leça do Balio, Portugal; hugo.carvalho@cedivet.pt (H.L.d.C.); goncalo.barros@cedivet.pt (G.B.); 2 Department of Veterinary Sciences, University of Trás-os-Montes e Alto Douro (UTAD), 5000-801 Vila Real, Portugal; 3 Department of Veterinary and Animal Sciences, CESPU University, 4585-116 Gandra, Portugal; 4 UNIPRO—Oral Pathology and Rehabilitation Research Unit, CESPU University, 4585-116 Gandra, Portugal; 5 AniCura Santa Marinha Veterinary Hospital, R. Dom Henrique de Cernache 183, 4400-625 Vila Nova de Gaia, Portugal; catia.fernandes@anicura.pt; 6 Cytology and Hematology Diagnostic Services, Laboratory of Histology and Embryology, Department of Microscopy, ICBAS-School of Medicine and Biomedical Sciences, University of Porto (U. Porto), Rua de Jorge Viterbo Ferreira, 228, 4050-313 Porto, Portugal

**Keywords:** abdominal effusion, case report, cell tube block, feline coronavirus, feline infectious peritonitis, Sysmex XN-1000V

## Abstract

Feline infectious peritonitis (FIP) can be overlooked in older cats with competing causes of effusion. An older female domestic shorthair cat with hyperthyroidism and a hypertrophic cardiomyopathy phenotype developed abdominal effusion in December 2023. Initial effusion total protein was 5.46 g/dL, the albumin-to-globulin ratio was 0.48 and delta total nucleated cell ratio (ΔTNC) was 60.58, yielding a retrospectively calculated FIP Effusion Index of 126.2. Coronavirus RT-PCR detected FCoV RNA, with M1058L mutation positivity, and cell tube block immunohistochemistry (CTB-IHC) demonstrated viral antigen within macrophages. A second sample collected from scant residual effusion 9 days after the initiation of GS-441524 had a ΔTNC of 2.15 and a FIP Effusion Index of 7.17, despite a higher total protein concentration and a lower albumin-to-globulin ratio. GS-441524 treatment began on 20 December 2023 and ended on 22 March 2024. Abdominal fluid was no longer detected at the assessment on 25 January. No confirmed FIP relapse was documented before death during cardiopulmonary decompensation in January 2026. This case illustrates complementary contributions of the FIP Effusion Index and CTB-IHC, with an exploratory observation of serial FIP Effusion Index change. Threshold comparison is retrospective. The lower index was observed during treatment alongside markedly reduced effusion, but this observation does not establish a treatment effect or validate treatment monitoring.

## Simple Summary

Feline infectious peritonitis can be difficult to recognise in older cats with other causes of fluid accumulation. An older female cat with thyroid and heart disease developed abdominal fluid. An initial FIP Effusion Index of 126.2 was consistent with the diagnosis, and cell tube block immunohistochemistry demonstrated coronavirus protein within macrophages. The FIP Effusion Index fell to 7.17 in a second sample collected from scant residual abdominal fluid 9 days after GS-441524 treatment began. The abdominal fluid subsequently resolved. The case illustrates complementary information from routine fluid measurements and cellular antigen detection. Serial FIP Effusion Index measurements provide an observation for future studies, although their ability to monitor treatment response remains unvalidated.

## 1. Introduction

Feline infectious peritonitis (FIP) is a systemic inflammatory disease associated with feline coronavirus (FCoV) infection, characterised by monocyte/macrophage involvement, vascular injury and variably distributed pyogranulomatous inflammation [1]. Increased vascular permeability can produce protein-rich abdominal, pleural or pericardial effusions [2,3]. Although FIP is strongly associated with young age, disease in an older cat remains possible [4]. Age therefore modifies clinical suspicion but cannot determine whether further investigation is warranted [3,4,5].

An effusion provides a relatively accessible specimen for assessing both the inflammatory response and the presence of FCoV. Protein concentrations, the albumin-to-globulin (A/G) ratio, cytomorphology and the Rivalta test contribute to the diagnostic assessment, whereas molecular assays and immunostaining provide evidence of the agent [1,3,6,7]. None of these findings should be interpreted without considering the sample type, clinical context and analytical limitations. This distinction is particularly relevant when signalment is atypical and alternative causes of abdominal effusion remain plausible.

Automated haematology analysers can provide an additional diagnostic clue through discordant nucleated-cell counts in different channels. Delta total nucleated cells (ΔTNC), initially investigated on the Sysmex XT-2000iV (Sysmex Corporation, Kobe, Japan) [8], have subsequently been assessed on the Sysmex XN-1000V (Sysmex Corporation, Kobe, Japan) [9,10,11,12]. Despite its name, ΔTNC is a dimensionless ratio. The FIP Effusion Index combines ΔTNC with the effusion A/G ratio [9,12]. These parameters support the selection and interpretation of further diagnostic tests, including molecular assays and cell tube block immunohistochemistry (CTB-IHC) [1,6,13,14,15,16,17,18,19,20,21,22].

This case report examines the complementary contributions of the FIP Effusion Index and CTB-IHC in an older cat with pre-existing cardiac and thyroid disease. Two effusion analyses performed before and during GS-441524 treatment provide an exploratory longitudinal observation. The clinical course, including GS-441524 treatment and prolonged follow-up, supplies context for interpreting the diagnostic findings.

## 2. Case Presentation

### 2.1. Clinical History and Initial Assessment

A 14-year-old female domestic shorthair cat with established hyperthyroidism and an echocardiographic hypertrophic cardiomyopathy phenotype was investigated for abdominal effusion in December 2023. Maintenance treatment included thiamazole, benazepril, furosemide and clopidogrel. Previous episodes of dyspnoea and pleural effusion in October 2023 had been managed in the context of cardiac disease. The pleural fluid was described as a non-septic exudate. At reassessment on 2 December, apparent weight gain was associated with free abdominal fluid. Abdominal ultrasonography identified no obvious mass, and thoracic radiographs showed a bronchointerstitial pattern. The abdominal effusion was classified as a non-septic exudate, prompting FCoV testing.

Subsequent molecular and serological findings, interpreted together with the compatible effusion phenotype and clinical presentation, supported a diagnosis of effusive FIP, which was recorded in the clinical record on 15 December. Progressive fluid accumulation required therapeutic abdominocentesis on 17 December, when approximately 250 mL was removed to improve comfort. On 19 December, the cat was hospitalised for marked lethargy and inadequate spontaneous food intake. Haematology showed anaemia (haematocrit, 23.7%; reference interval, 26–47%) and lymphopenia (0.27 × 10^9^/L; reference interval, 0.73–7.86 × 10^9^/L). The clinical record also noted Heinz bodies (3+) and toxic neutrophil changes. Serum albumin (2.6 g/dL), creatinine (0.8 mg/dL), alanine aminotransferase (14 U/L) and total bilirubin (0.2 mg/dL) were within the respective laboratory reference intervals. The essential clinical sequence is summarised in Table 1.

### 2.2. Effusion Biochemistry and Cytology

**Figure 1:**
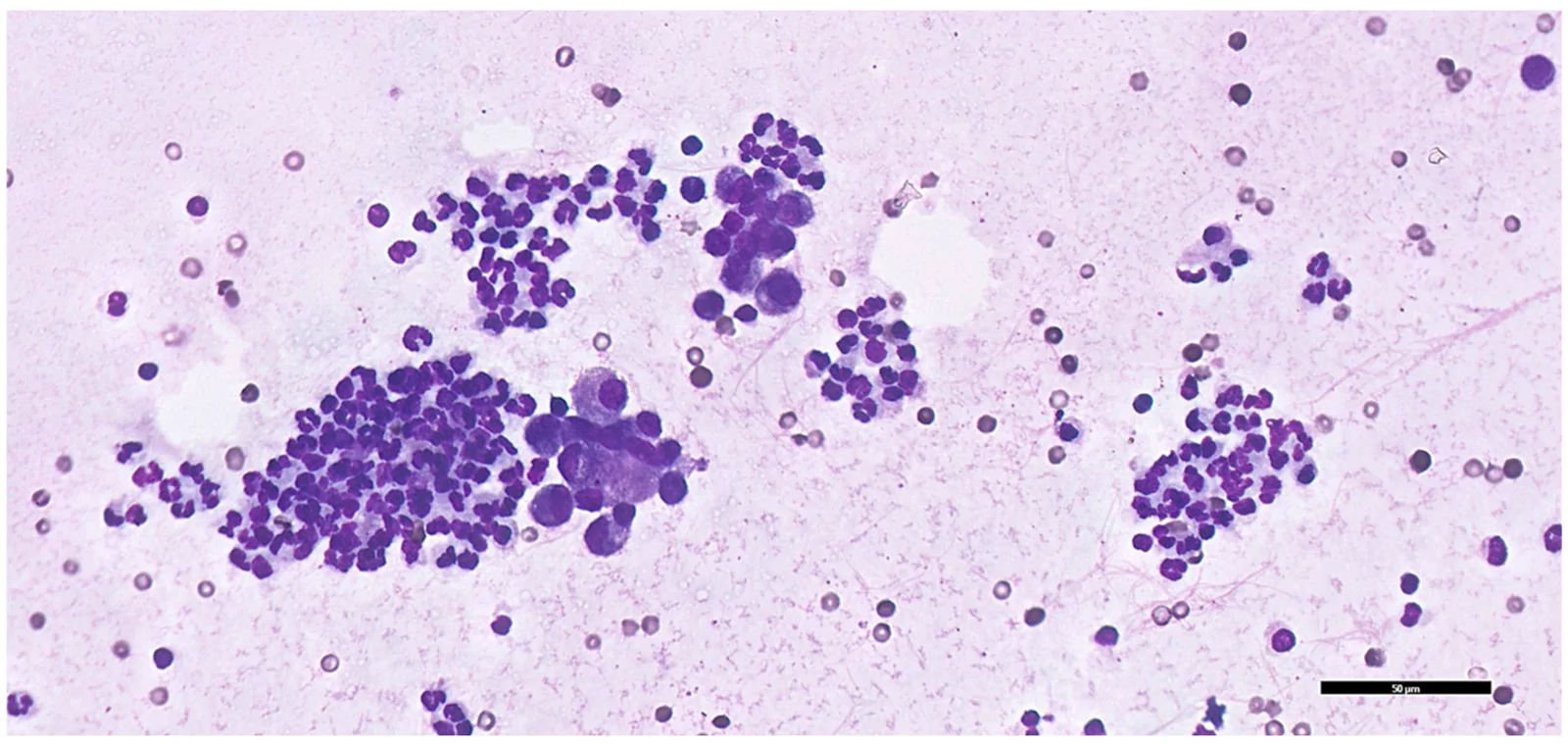
Cytology of the abdominal effusion showing activated macrophages and non-degenerate neutrophils in a proteinaceous background. May–Grünwald–Giemsa stain. Digital image acquired using Philips IntelliSite Pathology Solution 3.2. Scale bar = 50 μm.

Abdominocentesis yielded clear-to-yellow fluid, submitted in ethylenediaminetetraacetic acid (EDTA) and additive-free tubes. Initial effusion total protein was 5.46 g/dL, albumin was 1.78 g/dL and calculated globulins were 3.68 g/dL, giving an A/G ratio of 0.48. Protein measurements were performed on a Beckman Coulter AU680e analyser (Beckman Coulter, Cassina de’ Pecchi, Italy) and globulins were calculated as total protein minus albumin [9]. The Rivalta test, performed using the published acetic-acid precipitation method [9], was positive. Direct smears and cytocentrifuge preparations were stained with a Romanowsky-type May–Grünwald–Giemsa stain using a RAL Stainer (CellaVision RAL Diagnostics, Martillac, France). Activated macrophages predominated, with admixed non-degenerate neutrophils and occasional small, mature lymphocytes in a proteinaceous background (Figure 1). No microorganisms or overtly neoplastic cell populations were identified on cytological examination.

The protein-rich inflammatory effusion, low A/G ratio and positive Rivalta reaction supported investigation for FIP. Cytology alone did not establish the aetiology or exclude a poorly exfoliative neoplasm.

### 2.3. Automated Cell Counts and the FIP Effusion Index

**Figure 2:**
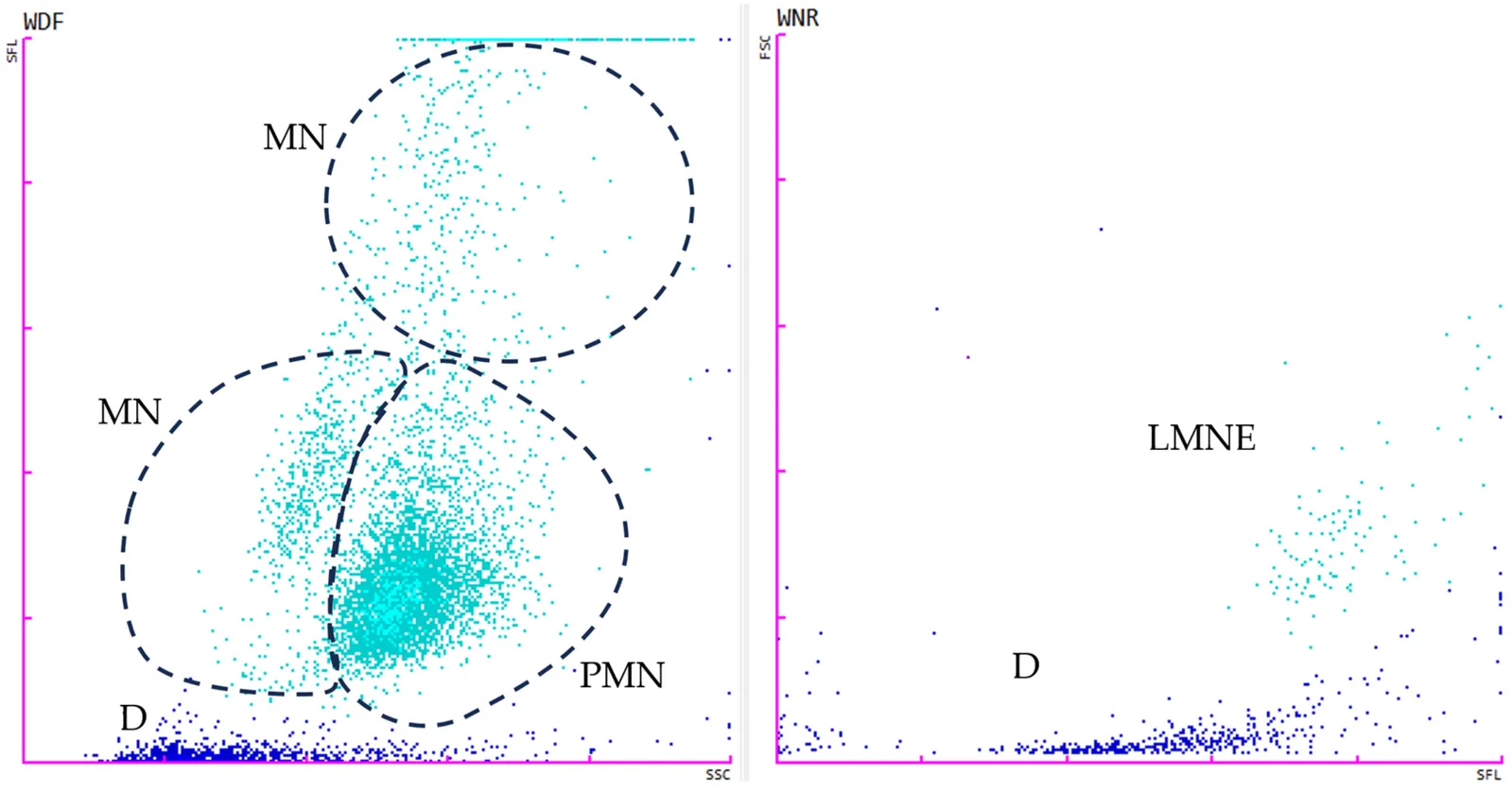
Sysmex XN-1000V scattergrams from the initial exudate analysed in whole-blood mode. The left panel shows the white blood cell differential (WDF) channel, from which TNCC-WDF was obtained, whereas the right panel shows the white cell nucleated (WNR) channel, from which TNCC-WNR was obtained. D (dark blue), debris; LMNE (light blue), leukocyte populations other than basophils; MN, mononuclear cells, including macrophages, monocytes and lymphocytes; PMN, polymorphonuclear cells, predominantly neutrophils. In this exudate, the marked discrepancy of nucleated-cell events between the WDF and WNR channels resulted in a high inter-channel ratio (ΔTNC = 60.58). TNCC, total nucleated cell count; ΔTNC, delta total nucleated cells.

The abdominal effusion collected in EDTA was analysed on a Sysmex XN-1000V (Sysmex Corporation, Kobe, Japan) in whole-blood mode. The initial total nucleated cell count (TNCC) obtained in the white blood cell differential (WDF) channel (TNCC-WDF) was 7267 cells/µL, whereas the TNCC obtained in the white cell nucleated (WNR) channel (TNCC-WNR) was 120 cells/µL, with a reported ΔTNC of 60.58 (Figure 2). In accordance with the published method, ΔTNC was defined as the dimensionless ratio TNCC-WDF/TNCC-WNR [9]:

$$∆\mathrm{TNC}=\frac{\mathrm{TNCC}-\mathrm{WDF}}{\mathrm{TNCC}-\mathrm{WNR}}$$

The FIP Effusion Index was then calculated by dividing ΔTNC by the effusion A/G ratio:

$$\mathrm{FIP Effusion Index}=\frac{∆\mathrm{TNC}}{A/\mathrm{G ratio}}$$

The initial FIP Effusion Index exceeded the threshold of 7.54 proposed in 2024 [9]. Because the clinical episode occurred in December 2023, comparison with the published thresholds represents retrospective interpretation rather than evidence that the FIP Effusion Index guided the original treatment decision. A second effusion sample, collected on 29 December 2023 from scant residual free abdominal fluid, 9 days after GS-441524 initiation, yielded ΔTNC of 2.15, total protein of 8.14 g/dL, albumin of 1.90 g/dL, globulins of 6.24 g/dL and A/G ratio of 0.30. The reported cellular count was 9117 cells/µL, and the Rivalta test was negative. Cytology identified non-degenerate neutrophils, activated macrophages and reactive mesothelial cells. The fluid was classified as a non-septic exudate. Both FIP Effusion Index values were calculated retrospectively from the reported ΔTNC and A/G ratios rounded to two decimal places: 60.58/0.48 = 126.2 and 2.15/0.30 = 7.17 (Table 2). The follow-up cellular count and ΔTNC were retained as reported; the original follow-up WDF and WNR channel outputs were unavailable for independent verification. The first sample was collected on 4 December 2023, before prednisolone was initiated on 15 December and GS-441524 on 20 December. The second sample was collected during both treatments.

### 2.4. Molecular Analysis and Cell Tube Block Immunohistochemistry

The effusion sample was submitted to IDEXX Laboratories (Barcelona, Spain) for FCoV RT-PCR testing. FCoV RNA was detected, with subsequent mutation analysis positive for M1058L and negative for S1060A. The laboratory report classified the detected virus as the feline infectious peritonitis virus (FIPV) biotype, as reported on 6 December 2023. The report did not provide a cycle threshold or quantitative viral load. Serum FCoV antibody testing was performed using a commercial enzyme-linked immunosorbent assay (BioPronix FCoV Ab ELISA, Agrolabo, Scarmagno, Italy). All tested dilutions, including 1:12,800, were positive on the same date. Serology was interpreted as evidence of FCoV exposure rather than confirmation of FIP.

**Figure 3:**
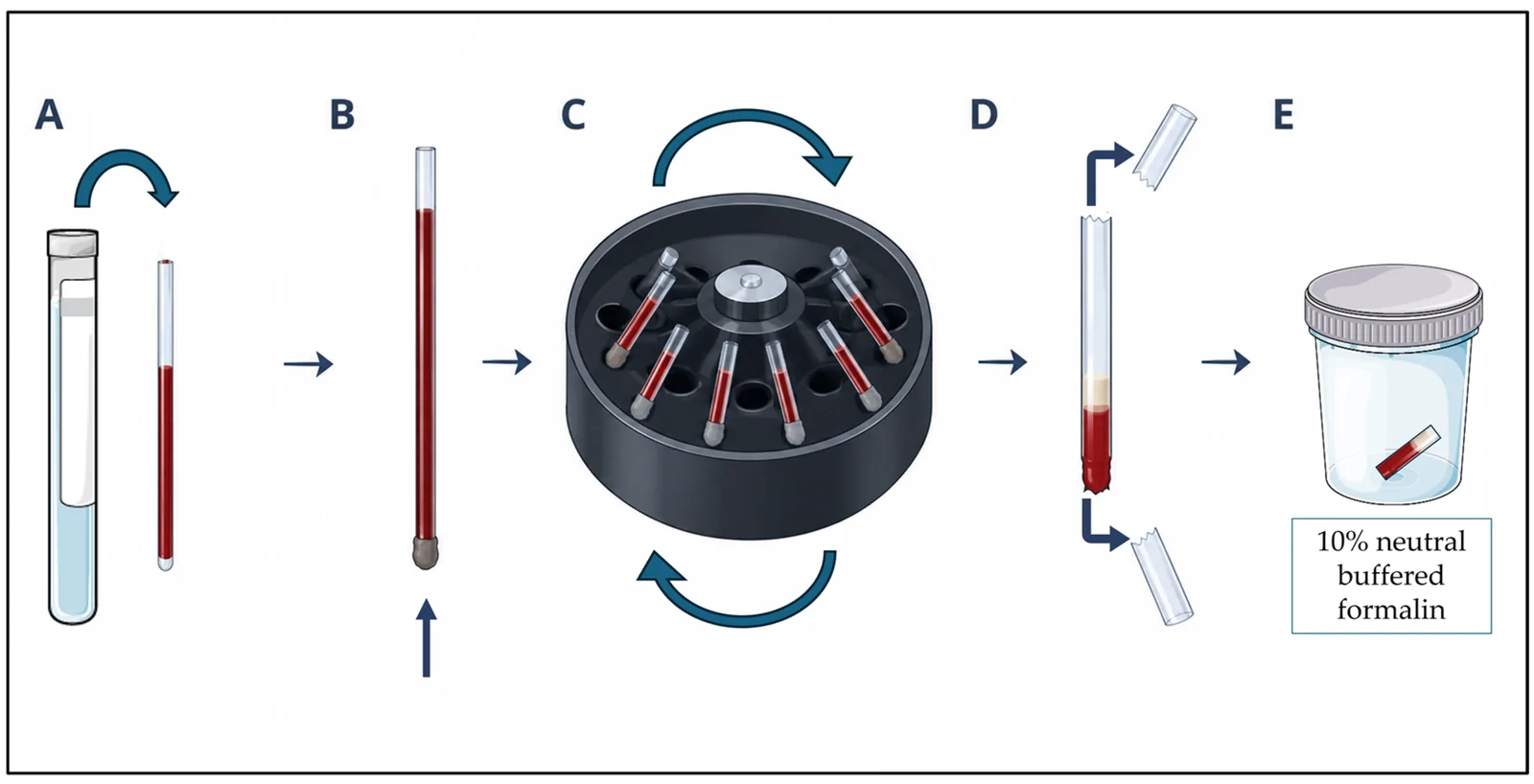
Schematic representation of the cell tube block technique. (**A**) Filling of the capillary tube with the fluid sample. (**B**) Sealing of the lower end of the capillary tube with clay. (**C**) Centrifugation of the filled capillary tube to concentrate cells at the buffy coat interface. (**D**) Cutting the capillary tube at both ends of the buffy coat to recover the concentrated cellular material. (**E**) Transfer of the recovered cellular material into 10% neutral buffered formalin for fixation.

Cell tube blocks were prepared using the CEDIVET protocol adapted from published CTB techniques [6,13,14]. Effusion samples were centrifuged at approximately 2665× *g* for 5 min. The resulting cell pellet was concentrated in haematocrit capillary tubes by repeat centrifugation, fixed in 10% neutral buffered formalin for 24 h and embedded in paraffin (Figure 3). The recorded 2665× *g* for 5 min refers to the initial sedimentation step. The relative centrifugal force and duration of the subsequent capillary-tube centrifugation were unavailable in the case-specific documentation.

**Figure 4:**
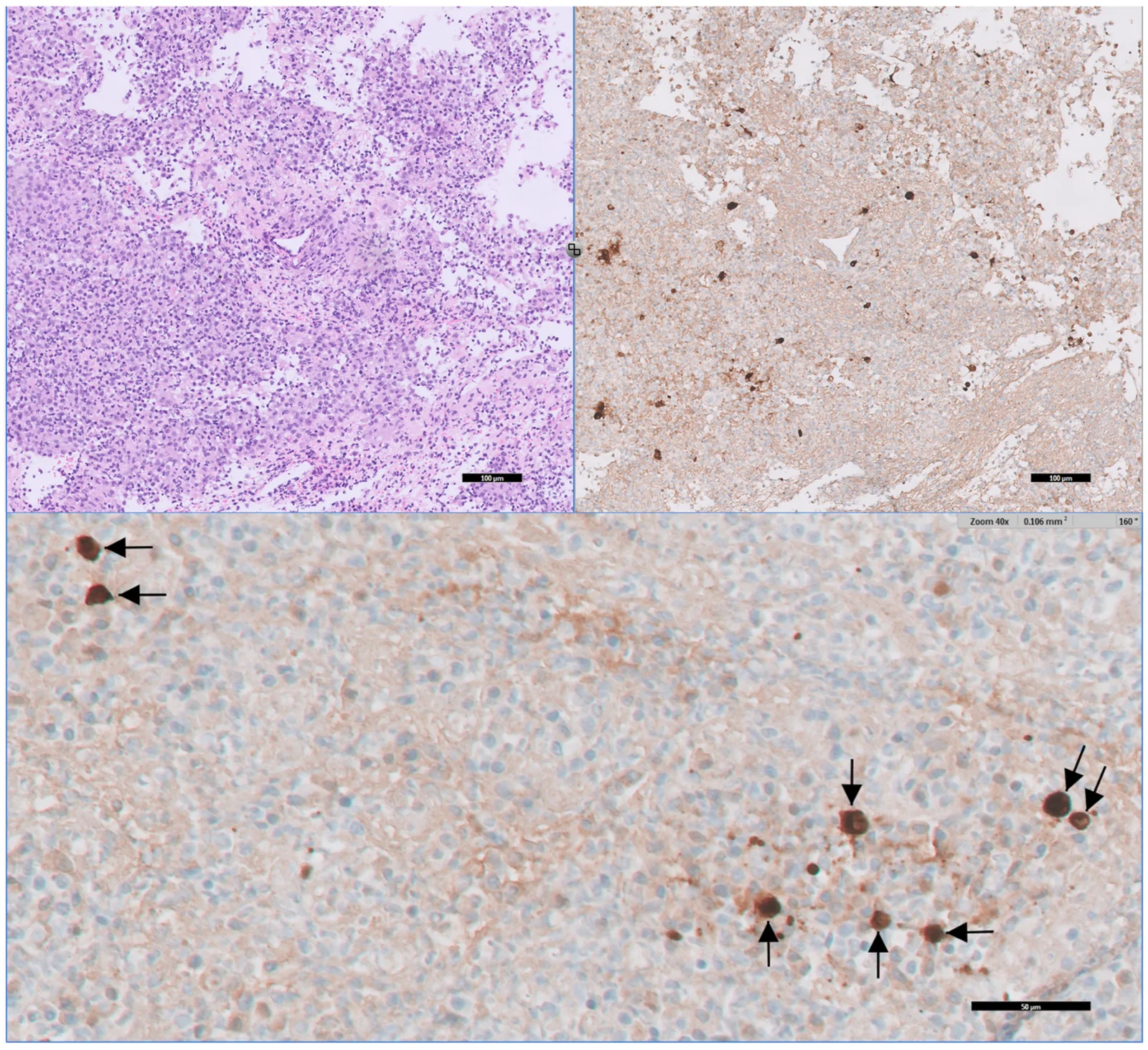
Abdominal effusion cell tube block. (**Upper left**): haematoxylin and eosin-stained section showing macrophage-rich inflammation with non-degenerate neutrophils. (**Upper right**): FCoV immunohistochemistry with monoclonal antibody clone FIPV3-70, demonstrating cytoplasmic immunoreactivity in macrophages. (**Lower panel**): higher-magnification view of immunoreactive macrophages (black arrows). Immunostaining was visualised using 3,3′-diaminobenzidine with haematoxylin counterstaining. Digital images were acquired using Philips IntelliSite Pathology Solution 3.2. Scale bars = 100 μm (**upper panels**) and 50 μm (**lower panel**).

Sections were cut at 3 µm and stained with haematoxylin and eosin. Immunohistochemistry used a VENTANA BenchMark ULTRA platform (Ventana Medical Systems, Tucson, AZ, USA) and the monoclonal anti-FCoV antibody clone FIPV3-70 (MA1-82189; Invitrogen, Thermo Fisher Scientific, Waltham, MA, USA), diluted at 1:100. Heat-induced antigen retrieval used ULTRA Cell Conditioning Solution at 96 °C for 32 min, followed by primary antibody incubation for 36 min at 36 °C. Signal was developed using 3,3′-diaminobenzidine (DAB) with the OptiView DAB IHC Detection Kit and haematoxylin counterstaining. The laboratory protocol included known FIP-positive tissue and omission of the primary antibody as positive and negative controls, respectively. Sections showed macrophagic–neutrophilic inflammation and granular cytoplasmic FCoV immunoreactivity within macrophages (Figure 4).

Compatible inflammatory cytology, low effusion A/G, FCoV RNA detection and intramacrophage antigen localisation supported effusive FIP. The initially elevated FIP Effusion Index provided concordant adjunctive information. No histopathological assessment of affected organs was performed because no accessible organ lesions suitable for biopsy or lymphadenomegaly were identified.

### 2.5. Treatment and Follow-Up

Treatment with GS-441524 (15 mg/kg once daily) began on 20 December 2023 and ended on 22 March 2024, approximately 13 weeks later. Treatment was initially subcutaneous, changed temporarily to oral administration on 24 February and returned to subcutaneous administration in March because the cat resisted oral medication. The dose remained 15 mg/kg once daily during oral administration. Supportive care included assisted feeding, mirtazapine and analgesia, alongside management of the comorbidities. Glucocorticoids were started before GS-441524 for suspected FIP-associated vascular inflammation and were tapered during the early treatment phase. Sublingual buprenorphine (0.02 mg/kg) was administered approximately 2 h before each injection to improve tolerance.

The FIP Effusion Index declined from 126.2 to 7.17 between 4 and 29 December 2023. At the second collection, 9 days after GS-441524 initiation, only scant residual free abdominal fluid remained. Clinical improvement was recorded by 8 January. On 20 January, haematocrit was 37.1% (reference interval, 24.0–45.0%), indicating resolution of the previously documented anaemia, although lymphopenia persisted (0.46 × 10^9^/L; reference interval, 1.5–7.0 × 10^9^/L). No free abdominal fluid was detected at the assessment on 25 January, 36 days after treatment began.

Serum protein electrophoretic separation was performed by capillary electrophoresis using the Sebia^®^ Minicap Flex Piercing Analyser (Sebia, Lisses, France), according to the manufacturer’s instructions. Serum electrophoretic A/G ratios were 0.74, 0.67 and 0.66 on 20 January, 26 February and 9 March, respectively, rising to 0.93 on both 29 April and 17 June (reference interval, 0.79–1.52). γ-Globulins were elevated in January, February and March (2.34, 1.98 and 2.25 g/dL, respectively), within the reference interval in April (1.82 g/dL) and mildly elevated again in June (2.10 g/dL; reference interval, 0.35–1.87 g/dL). α2-Globulins remained elevated in April and June (1.48 and 1.67 g/dL; reference interval, 0.54–1.35 g/dL).

Serum FCoV antibody titres were ≥1:12,800 in January, 1:1600 in February and again ≥1:12,800 in March and June. In April, serology was positive at the single reported dilution of 1:200. No confirmed clinical FIP relapse or additional antiviral course was documented. Weight loss prompted reassessment in October 2024. At that reassessment, serum α1-acid glycoprotein (AGP), measured by IDEXX Laboratories (Barcelona, Spain), was within the laboratory reference interval.

Cardiac and thyroid disease continued to require treatment, with recurrent pulmonary oedema and a stage C hypertrophic cardiomyopathy phenotype documented during later follow-up. On 16 January 2026, approximately 25 months after the FIP diagnosis and 22 months after antiviral treatment ended, the cat died following recurrent cardiopulmonary arrest during hospitalisation for respiratory decompensation, fever, hypotension, neutropenia and azotaemia. Serum albumin was 3.0 g/dL and globulins were 4.2 g/dL during the terminal admission, giving a reported A/G ratio of 0.7. No necropsy was documented. The terminal illness was not established as recurrent FIP.

## 3. Discussion

### 3.1. Diagnostic Reasoning in a Geriatric Cat with Comorbidities

This case illustrates the diagnostic difficulty of recognising FIP in an older cat with established explanations for fluid accumulation. Cardiac disease and hyperthyroidism were clinically relevant, but the inflammatory, protein-rich effusion warranted investigation beyond a solely cardiogenic mechanism. Although younger cats predominate in FIP cohorts [5], age should modify pre-test probability without excluding FIP when the fluid phenotype is compatible.

Contemporary treatment cohorts nevertheless include occasional geriatric cats. In a 307-cat cohort, age ranged up to 187 months (15.6 years), illustrating that advanced age cannot independently exclude FIP [4].

The abdominal imaging findings and absence of an overt neoplastic cell population reduced support for some competing diagnoses but could not exclude a poorly exfoliative tumour or concurrent disease [1]. Likewise, the earlier pleural effusion cannot retrospectively be attributed to FIP without corresponding agent-detection results. The diagnostic value of the abdominal sample lay in integrating inflammatory morphology with biochemical findings and direct evidence of FCoV.

### 3.2. Interpretation of the Effusion Phenotype

The initial high protein concentration, low A/G ratio and macrophage-rich inflammation were compatible with FIP but were not pathognomonic. The follow-up sample remained protein-rich and inflammatory, with reactive mesothelial cells also reported. The laboratory classification as a non-septic exudate described the fluid phenotype without establishing its cause.

The Rivalta test changed from positive initially to negative in the second sample. A retrospective diagnostic study reported sensitivity of 91.3% and specificity of 65.5% for FIP [23]. These estimates do not establish performance for treatment monitoring, and a negative result in the follow-up sample cannot be taken as proof of viral clearance. Serial changes require interpretation alongside cytology, protein measurements and the clinical course.

### 3.3. Meaning and Limitations of the Analyser-Derived Indices

ΔTNC reflects disagreement between analyser channels rather than a direct measurement of viral burden. Previous studies on the Sysmex XT-2000iV linked increased ratios to cell entrapment in precipitated material induced by acidic reagent [8]. A related explanation has been proposed for WNR undercounting on the XN platform [9,10].

A 2026 comparison of 45 feline effusions identified bias between ΔTNC values from the XT-2000iV and XN-1000V, despite concordant classification at the evaluated cut-offs [10]. Numerical interchangeability between platforms should not be assumed.

The original 50-sample study reported 96.3% sensitivity and 95.7% specificity at a threshold of approximately 5.06, and 96.3% sensitivity and 100% specificity at approximately 7.54 [9]. These estimates describe that study population rather than universal performance. The initial value of 126.2 exceeded both thresholds. The follow-up value of 7.17 lies within the published highly suggestive range, between 5.06 and 7.54. However, diagnostic thresholds have not been established as treatment targets, and crossing below 7.54 cannot define remission or justify stopping antiviral treatment.

The longitudinal findings are informative because the FIP Effusion Index fell between 4 and 29 December 2023 despite an increase in total effusion protein (5.46 to 8.14 g/dL) and a decrease in A/G ratio (0.48 to 0.30). Its decline was therefore driven by the reduction in reported ΔTNC (60.58 to 2.15), rather than by uniform biochemical improvement. The second sample was obtained from scant residual free abdominal fluid 9 days after GS-441524 initiation. The decline coincided with treatment, but two measurements in one cat receiving glucocorticoids and GS-441524 cannot establish a treatment effect or distinguish biological change from analytical or pre-analytical variation.

### 3.4. Viral Detection and the Practical Contribution of CTB-IHC

Effusion RT-PCR provided evidence of FCoV RNA in a compatible clinical setting [22]. The additional M1058L result was supportive, but spike mutation detection does not establish a uniquely FIP-specific viral pathotype [3,24]. Likewise, serum antibody titres indicate exposure and cannot independently confirm FIP or diagnose relapse [3,24]. The fluctuation in titres during follow-up therefore does not contradict the documented clinical improvement and should not be substituted for assessment of the cat.

Detection of M1058L added supportive molecular information but should not be interpreted as a pathognomonic FIP marker, because spike-mutation assays have not shown perfect discrimination between FIP and non-FIP populations [25].

CTB-IHC added cellular localisation to the molecular evidence by demonstrating FCoV antigen within morphologically identifiable macrophages. The CTB technique produces formalin-fixed, paraffin-embedded material from fluid cytology specimens, enabling morphological assessment and immunohistochemistry on serial sections [14]. Its practical contribution is the ability to demonstrate antigen within inflammatory cells using an accessible effusion sample, without obtaining an organ biopsy for that diagnostic step.

Interpretation requires unequivocal macrophage staining, morphological correlation and appropriate controls. Findings from conventional effusion immunocytochemistry [26] should not be assumed to apply identically to CTB-IHC, and performance depends on the specific protocol. A cell tube block preserves cellular detail but does not reproduce the tissue architecture of organ lesions. The FIP Effusion Index and CTB-IHC therefore answer different questions: the FIP Effusion Index supports the decision to investigate FIP, while CTB-IHC localises viral antigen within the inflammatory population.

### 3.5. Clinical Relevance and Limitations

Resolution of effusion during GS-441524 treatment and the absence of a confirmed relapse during prolonged follow-up were consistent with sustained clinical improvement without documented FIP recurrence [7,27,28,29]. Concurrent diuretic treatment and the short glucocorticoid course started while awaiting GS-441524 and tapered during early treatment limit the attribution of the early clinical response to GS-441524 alone. Serum A/G ratio subsequently returned to the reference interval, although α2-globulin elevation persisted and γ-globulins fluctuated. Recurrent hypergammaglobulinaemia and persistent seropositivity are nonspecific and did not establish treatment failure. The normal α1-acid glycoprotein result reported in October 2024 was reassuring in context but could not independently exclude relapse. Likewise, the terminal serum A/G ratio of 0.7 neither confirmed nor excluded recurrent FIP. The later cardiopulmonary deterioration cannot establish FIP recurrence or a disease-specific prognosis in the absence of a necropsy.

A large retrospective cohort of 307 treated cats further supports the possibility of sustained clinical remission after nucleoside-analogue therapy while also documenting that relapse can occur in a minority of cases [4].

More recent evidence has shown that a 42-day course of 15 mg/kg oral GS-441524 can be as effective as 84 days in selected cats with effusive FIP. However, differences in formulation, route, dose and study eligibility preclude retrospective application of that regimen to the present 2023 case [30].

Persistent or fluctuating FCoV antibody titres should not be interpreted as evidence of treatment failure in isolation, because transient titre increases and renewed faecal FCoV shedding have been documented in cats remaining in complete clinical remission after GS-441524 treatment [31].

Serum α1-acid glycoprotein has been shown to decrease rapidly during successful GS-441524 treatment, supporting its value as a contextual monitoring marker. However, a normal isolated value cannot independently establish remission or exclude relapse [32].

Limitations include the single-case design, absence of replicate FIP Effusion Index measurements and lack of organ histopathology or necropsy. The reconstructed follow-up index assumes the published ΔTNC convention. Follow-up relied on routine records rather than a standardised relapse protocol. The serial observations do not validate treatment monitoring, and the report cannot establish independent test accuracy, prospective decision impact or virological clearance.

## 4. Conclusions

In a geriatric cat with cardiac and thyroid disease, an initially markedly increased FIP Effusion Index was concordant with effusive FIP, while CTB-IHC localised FCoV antigen within macrophages and complemented molecular detection of viral RNA. A lower FIP Effusion Index was observed in a second sample obtained from scant residual abdominal fluid 9 days after GS-441524 initiation, providing an exploratory longitudinal observation that warrants further investigation. Following GS-441524 treatment, the abdominal effusion resolved and no confirmed FIP relapse was documented during long-term follow-up. This case highlights the value of integrating routine effusion analysis, analyser-derived parameters and cellular FCoV antigen detection within a multimodal diagnostic approach. Although the FIP Effusion Index may provide additional longitudinal information, its role in monitoring treatment response, defining remission or guiding treatment duration remains to be established.

## Figures and Tables

**Table 1:** Clinical timeline of the effusive episode, treatment and follow-up.

Date	Clinical Milestone
October 2023	Dyspnoea and pleural effusion with thyroid and cardiac disease. Fluid is described as a non-septic exudate.
2–19 December 2023	Initial abdominal effusion investigation; first sample collected on 4 December. FCoV testing reported on 6 December. FIP recorded and prednisolone initiated on 15 December, drainage on 17 December and hospitalisation on 19 December.
20 December 2023	Subcutaneous GS-441524 was initiated at 15 mg/kg once daily, with sublingual buprenorphine (0.02 mg/kg) administered approximately 2 h before each injection to improve tolerance.
29 December 2023	Repeat effusion sample collected from scant residual free abdominal fluid, 9 days after GS-441524 initiation.
January 2024	Clinical improvement by 8 January. Haematocrit within the reference interval on 20 January. No free abdominal fluid detected at the assessment on 25 January.
24 February–22 March 2024	Temporary change to oral GS-441524 treatment, then return to subcutaneous GS-441524 injections because of difficulty administering oral medication. Antiviral treatment was completed on 22 March.
April 2024–2025	Serum albumin-to-globulin ratio was 0.93 in April 2024, while FCoV antibody seropositivity persisted. Despite progressive weight loss noted in October 2024, α1-acid glycoprotein remained within the reference interval, and no recurrent effusion or other clinical findings supporting FIP relapse were documented. No confirmed FIP relapse was documented, while cardiac and thyroid disease continued to require management.
16 January 2026	Death during acute cardiopulmonary decompensation. No necropsy or confirmed FIP recurrence.

Calendar dates refer to the clinical history and laboratory reports. The effusion samples were collected on 4 and 29 December 2023, 25 days apart. Prednisolone was initiated on 15 December and GS-441524 on 20 December 2023; the repeat sample was obtained 14 and 9 days after initiation, respectively.

**Table 2:** Serial effusion findings before and during GS-441524 treatment.

Investigation	Initial Sample (4 December 2023)	Repeat Sample (29 December 2023)
Total protein (g/dL)	5.46	8.14
Albumin (g/dL)	1.78	1.90
Calculated globulins (g/dL)	3.68	6.24
Effusion A/G ratio	0.48	0.30
Rivalta test	Positive	Negative
Cellular count (cells/µL)	7267	9117
TNCC-WDF (cells/µL)	7267	9117
TNCC-WNR (cells/µL)	120	4240
Reported ΔTNC	60.58	2.15
FIP Effusion Index	126.2	7.17

All results refer to abdominal effusion. Samples were collected on 4 and 29 December 2023, 25 days apart. The repeat sample was collected 9 days after GS-441524 initiation (20 December) and 14 days after prednisolone initiation (15 December). Counts are expressed as cells/µL. ΔTNC and the FIP Effusion Index are dimensionless. Indices were calculated retrospectively using the reported ΔTNC and A/G ratios, rounded to two decimal places: 60.58/0.48 = 126.2 and 2.15/0.30 = 7.17. Using albumin/globulins directly gives 60.58/(1.78/3.68) = 125.2 and 2.15/(1.90/6.24) = 7.06. These are alternative calculations, not additional measurements. A/G, albumin-to-globulin ratio; FIP, feline infectious peritonitis; TNCC, total nucleated cell count; WDF, white blood cell differential channel; WNR, white cell nucleated channel.

## Data Availability

The data presented in this study are available upon request from the corresponding authors.

## Abbreviations

The following abbreviations are used in this manuscript:

A/G	Albumin-to-globulin ratio
AGP	α1-acid glycoprotein
CTB	Cell tube block
CTB-IHC	Cell tube block immunohistochemistry
ΔTNC	Delta total nucleated cells ratio
EDTA	Ethylenediaminetetraacetic acid
ELISA	Enzyme-linked immunosorbent assay
FCoV	Feline coronavirus
FIP	Feline infectious peritonitis
IHC	Immunohistochemistry
RT-PCR	Reverse transcription polymerase chain reaction
TNCC-WDF	Total nucleated cell count in the white blood cell differential channel
TNCC-WNR	Total nucleated cell count in the white cell nucleated channel
